# Variation partitioning in double-constrained multivariate analyses: linking communities, environment, space, functional traits, and ecological niches

**DOI:** 10.1007/s00442-021-05006-6

**Published:** 2021-08-11

**Authors:** Ioan Sîrbu, Ana Maria Benedek, Monica Sîrbu

**Affiliations:** 1 Faculty of Sciences, Lucian Blaga University of Sibiu, 5-7 Dr. I. Ratiu St., 550012 Sibiu, Romania; 2 Andrei Șaguna National Pedagogical College, 2 Aleea Turnu Roșu St., 550361 Sibiu, Romania

**Keywords:** dc-CA, Trait-based ecology, Ecological niche, Statistical graphics, Mollusk communities

## Abstract

Constrained multivariate analysis is a common tool for linking ecological communities to environment. The follow-up is the development of the double-constrained correspondence analysis (dc-CA), integrating traits as species-related predictors. Further, methods have been proposed to integrate information on phylogenetic relationships and space variability. We expand this framework, proposing a dc-CA-based algorithm for decomposing variation in community structure and testing the simple and conditional effects of four sets of predictors: environment characteristics and space configuration as predictors related to sites, while traits and niche (dis)similarities as species-related predictors. In our approach, ecological niches differ from traits in that the latter are distinguished by and characterize the individual level, while niches are measured on the species level, and when compared, they are characteristics of communities and should be used as separate predictors. The novelties of this approach are the introduction of new niche parameters, niche dissimilarities, synthetic niche-based diversity which we related to environmental features, the development of an algorithm for the full variation decomposition and testing of the community–environment–niche–traits–space (CENTS) space by dc-CAs with and without covariates, and new types of diagrams for the results. Applying these methods to a dataset on freshwater mollusks, we learned that niche predictors may be as important as traits in explaining community structure and are not redundant, overweighting the environmental and spatial predictors. Our algorithm opens new pathways for developing integrative methods linking life, environment, and other predictors, both in theoretical and practical applications, including assessment of human impact on habitats and ecological systems.

**Supplementary Information:**

The online version contains supplementary material available at 10.1007/s00442-021-05006-6.

## Introduction

**Fig. 1:**
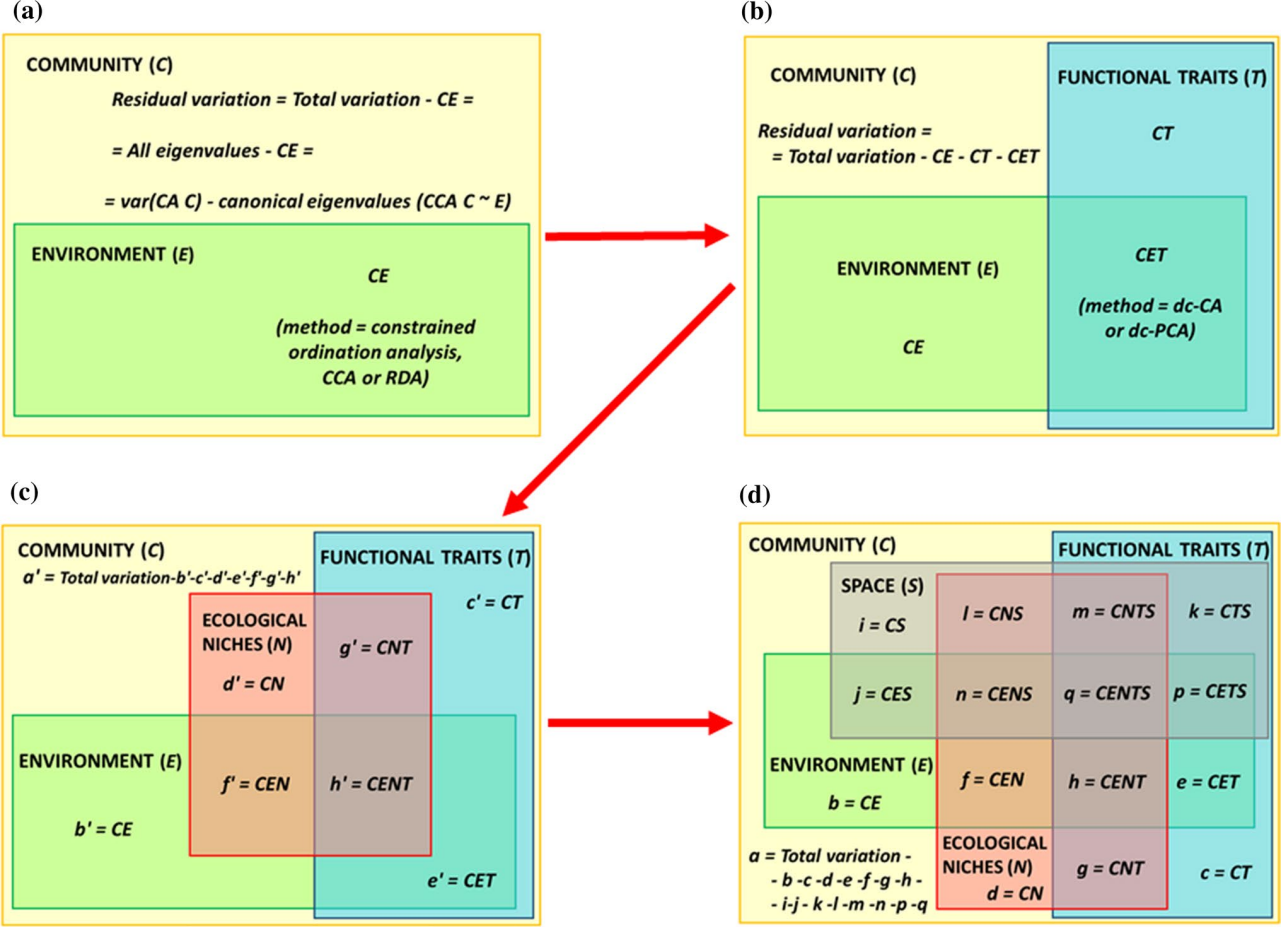
Diagram illustrating methodological evolution (from **a** to **d**) of integrating datasets to analyze relationships between communities and their predictors; (**c**) and (**d**) synthesize the gap that our article aims to fill: defining and analyzing the CENTS (community–environment–niche–traits–space) space by variation decomposition in double-constrained multivariate analyses (VADOC diagrams)

Describing, explaining, and modeling relationships between communities and their environment is the core of many modern ecological studies, aiming at answering a wide range of questions. From a methodological perspective, the number of datasets or matrices used in such studies has steadily increased (Legendre and Legendre 2012; ter Braak et al. 2018). The community matrix (C), usually a site-by-species table, and the unconstrained ordination analysis was expanded by adding a second table with environmental features (E) (Fig. 1a), used as predictors in constrained ordination (direct gradient) analysis. The addition of a third matrix, with functional traits (T) (Fig. 1b), aiming at testing hypotheses on how biological characteristics of species determine their responses to habitat features, was proposed, and methods of analysis were developed by several authors (Dolédec et al. 1996; Legendre et al. 1997; Dray and Legendre 2008; Lepš 2013; ter Braak et al. 2018; Peng et al. 2021; Pinho et al. 2021). This key approach evaluates processes, functions, and services of ecosystems (Céréghino et al. 2018; Sterk et al. 2013).

Until recently, correspondence analysis with linear external constraints on both rows and columns (Legendre and Legendre 2012) lacked full mathematical treatment and readily available algorithms and software. ter Braak et al. (2018) provided an explicitly defined mathematical algorithm for double-constrained correspondence analysis (dc-CA) and its linear counterpart, the double-constrained principal components analysis (dc-PCA), which are embedded in the software Canoco since version 5.10 (ter Braak and Šmilauer 2018). An R function for dc-CA was presented by ter Braak et al. (2018), with examples in Peng et al. (2021) and Pinho et al. (2021).

Another component in explaining the variability in community structure is space. One method to extract spatial patterns, which can be used as space predictors (S), from available spatial coordinates is the distance-based Moran eigenvector maps (db-MEM) (Borcard and Legendre 2002), formerly known as principal coordinates of neighbor matrices (PCNM), improved by Dray et al. (2006). Further, incorporating distances from phylogenetic trees became a standard routine to account for the so-called patristic relationships when investigating responses of species and their traits to environment gradients (Desdevises et al. 2003; de Bello et al. 2005, 2017; Pavoine et al. 2011).

Few methods have been developed so far that have taken an integrative approach to the analysis of the four datasets related to predictors of community assembly, and most of them include traits, phylogeny, environment, and space, relating them in various ways. Some of them aim to find spatial patterns in the components of trait diversity attributable to phylogenetic effects and environmental effects in the geographical space (Diniz-Filho et al. 2007). Others remove the phylogenetic signal in traits and the spatial information in environmental variables to associate ‘phylogenetic-free’ traits with ‘space-free’ environment, looking at the unique effects of environment and traits on species composition (Kühn et al. 2009). One of the oldest multivariate methods for trait–environment analysis, the RLQ, is also a three table-related problem (R stands for environment, L for community, and Q for traits matrices) aiming at estimating the parameters, by maximizing the covariance, in a fourth-corner matrix that crosses the habitat characteristics with the traits of species (Dray and Legendre 2008; Legendre and Legendre 2012). The RLQ is based on co-inertia analysis (Dray et al. 2003) and uses no regression (ter Braak et al. 2018). Pavoine et al. (2011) developed a statistical approach based on RLQ, which analyzes environmental filtering in an explicit geographic and phylogenetic context. However, although the abstract and some figures may suggest that variation partitioning was performed, RLQ is not suited for that goal because it is covariance based and does not use the same scale to measure variance in the different analyses (Peng et al. 2021). Also, RLQ requires a scaling method to make the predictor matrices better comparable and neither tests nor explicitly selects traits or environment predictors. In contrast, with dc-CA one can test and select both traits and environmental variables. In addition, dc-CA is scale invariant, so that no scaling method needs to be selected. It also allows variation partitioning, because it is regression based and its explained variance is in terms of inertia of the abundance table. Peng et al. (2021) suggested the possibility of variation decomposition in dc-CA, and Pinho et al. (2021) partitioned the trait-structured variation (in our approach, this being the blue part in Fig. 1) in neotropical forest composition using dc-CA, showing the unique and shared effects of climate and geographic position. This approach can be used analogously to decompose the environmentally structured variation (the green part in Fig. 1) based on two sets of predictors related to species by transposing the community matrix. Here, we propose an algorithm for decomposing the total variation in community composition (and not only the trait- or environmentally structured variation) into the parts attributable to four sets of predictors, i.e., two related to sites (environment and space) and two related to species (functional traits and ecological niche).

The concept of ecological niche is considered of paramount importance in understanding life–environment relationships (Krebs 2014) while being also subject to a wide array of concepts and measurements, but sometimes affected by ambiguity or equivocal use (Peterson and Soberón 2012; Soberón et al. 2017; Melo-Merino et al. 2020). In multivariate analyses, niches were mostly defined by functional traits (Violle and Jiang 2009; Kearney et al. 2010). Thus, traits and niches have been used interchangeably, or traits have been considered a proxy for assessing niches and included as such in constrained analyses. In our approach, we consider that niches, especially their overlap (or similarity), should be used separately from traits because they relate to different aspects concerning the species and communities. The difference in the species niches characterizes the outcome of their phylogeny, adaptations, and relationships with both biotic and abiotic factors. This synthetic information is contained in the niche dissimilarity matrix and cannot be sufficiently represented by individual (and often related) traits. We consider that another difference is that traits are distinguished and measured on the individual level, but niches can be characterized only at the species level and can be understood when comparing populations (species), revealing their meaning at the community level.

This paper defines and methodologically highlights the CENTS space, the acronym coming from *C*ommunity—*E*nvironment—*N*iche—(functional) *T*raits—*S*pace (Fig. 1). The first objective was to define, measure, and partition the CENTS space (Fig. 1d). Because spatial variability may not always be of interest, we also considered the CENT space (Fig. 1c) and, without a second set of species predictors, the CEST space. We propose an algorithm that combines three existing and widely used methods, the canonical correspondence analysis (CCA) or its linear counterpart, the redundancy analysis (RDA), dc-CA or its linear counterpart, dc-PCA, and the variation partitioning (VP) procedure (Borcard et al. 1992; ter Braak et al. 2018; ter Braak and Šmilauer 2018). The algorithm aims to disentangle and quantify the overlapping effects of E–S and T–N variable groups on C. Analyzing the CENTS space will considerably strengthen and link the fields of functional and structural ecology, thereby generating new insights and leading to a better understanding and quantification of the mechanisms underlying species–trait–environment patterns. In addition, the algorithm we developed may be used for other predictor data tables, such as a table with ecological indicator values or with phylogenetic relationships, and it also may be extended to include more than two data tables for sites or species.

Our second objective was to summarize how species relate to resources and their availability in the environment, synthesize this information in a standardized way, and use these novel measures to apply the algorithm mentioned above, including an N data table, measuring the ecological niche features of the species. For this goal, we proposed a new standardized metric of niche complementarity (dissimilarity) for both categorical and continuous resources, which also account for the availability of resources in the environment. We used this metric to define and measure the species’ uniqueness and one more aspect of the community diversity, the niche-based diversity (ND). We explored relationships between diversity measures and environment predictors, highlighting the use of ND in human impact assessment.

## Materials and methods

### Algorithm for variation decomposition and testing of CENT and CENTS space

To explain and decompose the variation in species composition, the algorithm uses the following tables:

i. C, the sites-by-species (occurrence, abundance or dominance) table, representing community structure. To study whether traits (or niche or both) can explain variation in species composition, one also needs the transpose of C, denoted by C’.
  All the other data tables considered in our algorithm include predictors (explanatory variables):
ii. E, the sites-by-environmental variables data table with values of environment descriptors of interest for each site in C.
iii. T, the species-by-traits data table with values of functional traits of each species in table C. Categorical traits can be expanded to sets of dummy (1/0) variables if needed. If the traits are a mix of distinct types (e.g., numeric, nominal, multi-choice, circular), the matrix T can be replaced by a species-by-species matrix of distances, for instance, using the Gower distance.
iv. N, a species-by-niche metrics table or a species-by-species table that describes the ecological niches of species. In other studies, N may contain tolerances to environment factors, bioindicator responses, or other features (except for traits). The niche overlap metrics may be based on any categorical or continuous resources (details in the section on niche measures).
v. S, a table describing the spatial configuration of the sites. It is based on coordinates or other variables indicating the position of the sites (e.g., latitude, longitude, altitude, depth), which are subject to a db-MEM analysis with forward selection (FWS) of predictors. In other studies, if S is a rectangular matrix and certain spatial descriptors (e.g., altitude) are of interest, it might be used as such.

Using these five data tables, we propose an algorithm for linking them and exploring their relationships. C plays the central role (of response variables), and there are two groups of predictors: group 1, comprising E + S, which apply to sites, being related indirectly to species through their distribution in sites, and group 2, comprising T + N, directly related to species and indirectly to sites through the structure of the communities they shelter.

In our algorithm, we used dc-CA because C is a compositional table and the length of the gradient was greater than 3, but when the axes are shorter, or the data are not compositional, the linear form (dc-PCA) might be used instead.

To apply dc-CA, the data sources that are not yet rectangular matrices (units by variables), such as (dis)similarity matrices, need first to be preprocessed to become rectangular. For this, the principal coordinates analysis (PCO) is used. Also, as dc-CA is a regression method, the number of row and column predictor variables should be kept small compared to the number of sites and species, respectively. To limit the number of predictors, we propose to use FWS of variables. In our algorithm, all variables within each group are denoted by the subscript (E_all,_ S_all_, T_all_, and N_all_), while the selected variables by FWS, are referred to without subscript.

Table 1 shows the preliminary steps needed when space predictors are included (CENTS space), the niche data is a (dis)similarity matrix, the number of environmental variables is large compared to the number of sites, and the number of traits is large compared to the number of species. When this is not the case, these preliminary steps (or some of them) are skipped.

**Table 1:** Preliminary steps for selecting variables and preparing the data for the algorithm of double-constrained analysis with variation partitioning

Step	Method	Response variables	Predictors group 1 (E, S)	Predictors group 2 (T, N)	Output and meaning
1	PCO	DHS			N_all_, i.e., PCO scores for the niche
2	VP dbMEM FWS	C	E_all_ and Coord		S_all_, i.e., spatial db-MEM eigenvectors (PCO scores), selection of S and E, and testing their simple effects
3	dc-CA FWS	C	E_all_	T_all_	Selection of T and verification of selection (or reaching consensus) for E
4	dc-CA FWS	C	E_all_	N_all_	Selection of N and verification of selection (or reaching consensus) for E

*E* environmental variables, *S* space variables, *T* traits, *N* niche variables, *C* community composition matrix, *PCO* principal coordinates analysis, *dbMEM* distance-based Moran Eigenvector Maps, *DHS* matrix of the standardized measure of niche dissimilarity between species, *Coord* geographical coordinates, *VP* variation partitioning procedure, *FWS* forward selection procedure, *dc-CA* double-constrained correspondence analysis. Subscript *all* indicates that all predictors of a group are included in the analysis. Terms with no subscript refer to the variables selected during FWS

### Estimating and testing the variation parts

Traditional direct ordination method (canonical ordination analysis) with one set of predictors (e.g., E) decomposed the green part in Fig. 1a into two parts: variation in species composition (C) explained by the environmental predictors (CE) and residual variation (variation of C not explained by E). The last is calculated by the difference between the total variation or all eigenvalues in C, assessed by a correspondence analysis of C (CA C), from which the explained variance (the canonical eigenvalues) obtained in the CCA of C constrained by E is subtracted. If two groups of predictors are used (tables with explanatory variables), either for species, for sites, or both (Fig. 1b shows the dc-CA parts of explained variance in C, using T and E as predictors), with the residual variation this gives four parts. Besides the explained variance assessment, the effects of the predictors—simple, conditional (unique), and shared—are also tested for their significance, against the null hypothesis that their effect is random. We have used the Monte Carlo permutation test, with 999 unrestricted permutations. Analogously, there are eight parts in CENT space analysis, which we coded with letters from *a'* to *h'* (Fig. 1c), but only some parts (or sum of parts) could be tested. The full decomposition of CENTS space gives 16 variation parts coded with letters from *a* to *q* (Fig. 1d).

In the following text, we refer to double-constrained analyses by their name (e.g., dc-CA C ~), where ~ is followed by the tables with the selected predictors. The symbol × is used to separate two groups of predictors, the first related to sites (E or S or both) and the second to species (T or N or both), while + indicates the use of variables belonging to both data tables in the group. Predictors from a table used as covariates are denoted by placing table abbreviation after the | (i.e., vertical line) symbol. For instance, the expression dc-CA C ~ (E + S) × (T|N) means that a double-constrained analysis is performed on the species-by-sites data table (C), with sites constrained by variables from combined E and S, and species constrained by T, while variables in N table act as covariates for the effects of T.

The decomposition of the community space using dc-CA is based on the idea that the canonical (double-constrained) eigenvalues resulting from this analysis represent the intersection between the effects of the environment (i.e., E + S) and traits (i.e., T + N) on species composition. In the VP, the explained variation is decomposed in the two conditional effects of the two predictors (or, more often, groups of predictors) and their overlap. Simple effects can be measured and tested using dc-CAs without covariates, while the conditional effects can be measured and tested using the analyses with covariates. For instance, when considering only the CENT space, analyses dc-CA C ~ (E) × (T), dc-CA C ~ (E) × (N), dc-CA C ~ (E) × (T + N) are used to separate the variation explained by T and N, when C is also constrained by E, and to test the simple effects. The explained variation in dc-CA C ~ (E) × (T + N) is the total explained variation (given by the sum of the conditional effect of T, N, and the shared variation), i.e., *e*' + *f*' + *h*' (Fig. 1c). In dc-CA C ~ (E) × (T), the explained variation is the simple effect of T (conditional effect of T and the overlap, i.e., *e*' + *h*'), and similarly, in dc-CA C ~ (E) × (N), it is the simple effect of N (conditional effect of N and the overlap, i.e., *f*' + *h*'). The significance for each of the simple effects is given by the double-constrained test on all ordination axes performed during the analyses without covariates. When only one dominant gradient is expected in the data, the significance of the test on the first axis will be considered. To test the conditional effects, we use dc-CA C ~ (E) × (T|N) (giving the conditional effect of T and its significance, i.e., *e*') and dc-CA C ~ (E) × (N|T) (giving the conditional effect of N and its significance, i.e., *f*'). The overlap (*h*') cannot be tested (Table 2).

**Table 2:** Algorithm for variation partitioning of CENT space relating communities (C) to the environment (E), traits (T), and ecological niches (N)

Step	Analysis	Estimates	Significance tests (*p*)
0	CA C	Total variation = All_eg	
1.1'	dc-CA C ~ (E) × (T|N)	*e*' = ceg[(E) × (T|N)] *c*' = ceg[T|N] − *e*'	{*b*' + *e*' + *f*' + *h*'} {*c*' + *e*'} {*e*'}^dc^
1.2'	dc-CA C ~ (E) × (N|T)	*f*' = ceg[(E) × (N|T)] *d*' = ceg[N|T] − *f*'	{*d*' + *f*'} {*f*'}^dc^
1.3'	dc-CA C ~ (E) × (T + N)	*h*' = ceg[(E) × (T + N)] − *e*' − *f*' *b*' = ceg[E] − ceg[(E) × (T + N)] *g*' = ceg[T + N] − ceg[(E) × (T + N)] − *c*' − *d*' *a*' = All_*eg* − *b*' − *c*' − *d*' − *e*' − *f*' − *g*' − *h*'	{*c*' + *d*' + *e*' + *f*' + *g*' + *h*'} {*e*' + *f*' + *h*'}^dc^
1.4'	dc-CA C ~ (E) × (T)		{*c*' + *e*' + *g*' + *h*'} {*e*' + *h*'}^dc^
1.5'	dc-CA C ~ (E) × (N)		{*d*' + *f*' + *g*' + *h*'} {*f*' + *h*'}^dc^

The tilde ~ stands for canonical ordination analysis, followed by the predictors placed in round brackets, separated by the × symbol, for discriminating between predictors related to sites (E) and those linked to species (T or N or both). The total variation (all eigenvalues, coded as All-eg) is given by the correspondence analysis of C (CA C). Canonical eigenvalues are coded as ceg, and the predictors used are enclosed in brackets []. Vertical bar | separates the covariates (to the right of it), the lowercase letters correspond to the variation parts given in Fig. 1c. Significance tests are related to the variation parts given in braces {} for the CCA and {}^dc^ (with superscript) for the dc-CA, also using the formerly mentioned letters

To measure all the elements of the community space and to test all the parts that may be tested, we not only need the synthetic results of the dc-CAs, but also the results of the two CCA (or RDA in dc-PCA) testing the constraints either on sites (with E or S or both as predictors, i.e., the canonical eigenvalues for site-related predictors only) or on species (with T or N or both as predictors, i.e., the canonical eigenvalues for species-related predictors only). These also allow the additional testing of some CENT (and CENTS) element sums. The variation parts of the community space can be calculated from various combinations of dc-CAs with covariates and without covariates, used in turn. The unadjusted percentages of explained variation (*R*^2^) will then be calculated by dividing the various variation parts by the total variation in the community composition, extracted from the species-by-sites table in the unconstrained CA (or PCA). The adjusted *R*^*2*^ can also be calculated (Peres-Neto et al. 2006; for details concerning dc-CA see ter Braak and Šmilauer 2018 and the online support site for Canoco 5.1).

We present the application of our algorithm using Canoco 5.12 software (ter Braak and Šmilauer 2018). At present, this resource has certain advantages over other softwares. It performs dc-CA with and without covariates, with optional forward selection of predictors, testing unique and shared effects, performs dimensionality tests, and illustrates results by double-constrained ordination diagrams. In addition, for a double-constrained analysis, Canoco also presents the intermediate results, i.e., of the two CCAs (or PCAs), testing the constraints on sites and separately on species. We also propose an alternative, Canoco independent algorithm, without covariates and CCAs included as separate steps, given in the Online Resource. This algorithm was inspired by the R-code for dc-CA variation partitioning in Pinho et al. (2021).

The algorithm for the decomposition of CENT space and testing its variation parts are explicitly given in Table 2, and the algorithm for CENTS is shown in Table 3. In some studies, ecological niche data are not available or of no research interest; therefore in the Online Resource, we provide the algorithm for CEST (community–environment–space–traits), with and without covariates, and illustrates the results using a novel graphical display, because the classical Venn diagrams proved to be inefficient. We call the visualization of these variation partitioning results VADOC diagrams (a term derived from ‘variation partitioning in double-constrained ordination analyses with multiple predictor tables diagrams’). In every algorithm, step 0 represents the unconstrained correspondence analysis of C (CA C), which returns the total variation in the response variables table, meaning the total eigenvalues.

**Table 3:** Algorithm for variation partitioning of CENTS space relating communities (C) to the environment (E), space (S), traits (T), and ecological niches (N)

Step	Analysis	Estimates	Significance tests (*p*)
0	CA C	Total variation = All_eg	
1.1	dc-CA C ~ (E|S) × (T|N)	*e* = ceg[(E|S) × (T|N)]	{*b + e + f + h*} {*c + e + k + p*} {*e*}^dc^
1.2	dc-CA C ~ (E|S) × (N|T)	*f* = ceg[(E|S) × (N|T)]	{*d + f + l + n*} {*f*}^dc^
1.3	dc-CA C ~ (E|S) × (T + N)	*b* = ceg[E|S] − ceg[(E|S) × (T + N)] *h* = ceg[E|S] − *b* − *e* − *f*	{*c + d + e + f + g + h + k + l + m + n + p + q*} {*e + f + h*}^dc^
1.4	dc-CA C ~ (E|S) × (T)		{*c + e + g + h + k + m + p + q*} {*e + h*}^dc^
1.5	dc-CA C ~ (E|S) × (N)		{*d + f + g + h + l + m + n + q*} {*f* + *h*}^dc^
2.1	dc-CA C ~ (S|E) × (T|N)	*k* = ceg[(S|E) × (T|N)]	{*i* + *k* + *l* + *m*} {*k*}^dc^
2.2	dc-CA C ~ (S|E) × (N|T)	*l* = ceg[(S|E) × (N|T)]	{*l*}^dc^
2.3	dc-CA C ~ (S|E) × (T + N)	*i* = ceg[S|E] − ceg[(S|E) × (T + N)] *m* = ceg[S|E] − *i* − *k* − *l*	{*k* + *l* + *m*}^dc^
2.4	dc-CA C ~ (S|E) × (T)		{*k* + *m*}^dc^
2.5	dc-CA C ~ (S|E) × (N)		{*l* + *m*}^dc^
3.1	dc-CA C ~ (E + S) × (T|N)	*c* = ceg[T|N] − ceg[(E + S) × (T|N)] *p* = ceg[T|N] − *c* − *e* − *k*	{*b* + *e* + *f* + *h* + *i* + *j* + *k* + *l* + *m* + *n* + *p* + *q*} {*e* + *k* + *p*}^dc^
3.2	dc-CA C ~ (E + S) × (N|T)	*d* = ceg[N|T] − ceg[(E + S) × (N|T)] *n* = ceg[N|T] − *d* − *f* − *l*	{*f* + *l* + *n*}^dc^
3.3	dc-CA C ~ (E + S) × (T + N)	*j* = ceg[E + S − ceg[(E + S) × (T + N)] − *b* − *i* *g* = ceg[T + N] − ceg[(E + S) × (T + N)] − *c* − *d* *q* = ceg[(E + S) × (T + N)] − *e* − *f* − *h* − *k* − *l* − *m* − *n* − *p* − *q* *a* = All_eig − *b* − *c* − *d* − *e* − *f* − *g* − *h* − *i* − *j* − *k* − *l* − *m* − *n* − *p* − *q*	{*e* + *f* + *h* + *k* + *l* + *m* + *n* + *p* + *q*}^dc^
3.4	dc-CA C ~ (E + S) × (T)		{*e* + *h* + *k* + *m* + *p* + *q*}^dc^
3.5	dc-CA C ~ (E + S) × (N)		{*f* + *h* + *l* + *m* + *n* + *q*}^dc^
4.1	dc-CA C ~ (E) × (T|N)		{*b* + *e* + *f* + *h* + *j* + *n* + *p* + *q*} {*e* + *p*}^dc^
4.2	dc-CA C ~ (E) × (N|T)		{*f* + *n*}^dc^
4.3	dc-CA C ~ (E) × (T + N)		{*e* + *f* + *h* + *n* + *p* + *q*}^dc^
4.4	dc-CA C ~ (E) × (T)		{*e* + *h* + *p* + *q*}^dc^
4.5	dc-CA C ~ (E) × (N)		{*f* + *h* + *n* + *q*}^dc^
5.1	dc-CA C ~ (S) × (T|N)		{*i* + *j* + *k* + *l* + *m* + *n* + *p* + *q*} {*k* + *p*}^dc^
5.2	dc-CA C ~ (S) × (N|T)		{*l* + *n*}^dc^
5.3	dc-CA C ~ (S) × (T + N)		{*k* + *l* + *m* + *n* + *p* + *q*}^dc^
5.4	dc-CA C ~ (S) × (T)		{*k* + *m* + *p* + *q*}^dc^
5.5	dc-CA C ~ (S) × (N)		{*l* + *m* + *n* + *q*}^dc^

The tilde ~ stands for canonical ordination analysis, followed by the predictors placed in round brackets, separated by the × symbol, for discriminating between predictors related to sites (E or S or both) and those linked to species (T or N or both). The total variation (all eigenvalues, coded as All-eg) is given by the correspondence analysis of C (CA C). Canonical eigenvalues are coded as ceg, and the predictors used are enclosed in brackets []. Vertical bar | separates the covariates (to the right of it), the lowercase letters correspond to the variation parts given in Fig. 1d. Significance tests are related to the variation parts given in braces {} for the CCA and {}^dc^ (with superscript) for the dc-CA, also using the formerly mentioned letters

The algorithm for decomposition of the CENTS space (Table 3) comprises five steps (1–5) in addition to step 0. In the first two steps, the variation in the community constrained by the unique effects of E and S (with S covariate in step 1 and E covariate in step 2) is decomposed. Step 3 decomposes the variation in the community constrained both by E and S, while in the last steps, the community is constrained either by E (step 4) or S (step 5). Each step has five substeps (#.1–#.5), homologous with the five steps (any or each denoted by #). Thus, the first two substeps are represented by dc-CAs in which the unique effects of T and N (T in substep #.1 and N in substep #.2) are included. Substep #.3 are dc-CAs with T + N as species-related predictors, while in the last two substeps T (substep #.4) and N (substep #.5) are included separately. For the calculation of CENTS elements, one needs only 9 of the total 25 substeps (in Table 3 one of the possible combinations of subsets is given), but the rest are needed to test various variation parts. For CENT space, one step with five substeps is needed (1.1'–1.5'), with only three substeps used to calculate variation parts (Table 2).

The formulas for calculating the variation parts involve the sum of canonical eigenvalues (coded as ceg) of the constrained analysis of predictor tables enclosed in brackets []. When only one group of predictors is used, the analysis is a CCA, and if both groups are present (separated by ×), the eigenvalues refer to the dc-CA. For instance, b' = ceg[E]-ceg[(E) × (T + N)] (step 1.3' in Table 2) means that the variation fraction termed b' in the CENT (Fig. 1c) is given by the subtraction of the sum of canonical eigenvalues of the double-constrained analysis involving E and (T + N) as predictors (dc-CA C ~ E × (T + N)), from the sum of canonical eigenvalues of the community constrained by the environmental variables (CCA C ~ E). Although the variation parts may be calculated and tested using several different combinations of analyses, only one is given in the proposed algorithm to avoid redundancy.

### Niche metrics

To explain the structure and functions of communities, independently of their species’ traits, to link the five matrices described in Fig. 1d and describe the niches' properties in terms of dissimilarities, differences, and diversity, we also developed some new niche metrics. These are based on a niche overlap index we developed earlier (Sîrbu 2006), which includes both the resource use by species and their availability in the environment and varies between 0 (completely distinct niches) and 1 (identical niches). Since, for our purposes, we found no suitable measure in the literature for fulfilling these requirements, we defined the HS_ij_, as the standardized niche overlap or similarity index (*H* is in honor of Hurlbert, while *S* stands for Smith) between the species *i* and *j*:

1 $${HS}_{i,j}=\frac{\sum_{k}{a}_{k}\sqrt{{p}_{i,k}{p}_{j,k}}}{\sqrt{\left(\sum_{k}{a}_{k}{p}_{i,k}\right)\left(\sum_{k}{a}_{k}{p}_{j,k}\right)}}$$

where *i* and *j* are indices of summation from 1 to *s* (number of species in the community), *k* is the index of summation from 1 to *n* (number of resources exploited), *p*_*ik*_ is the proportion of the species *i* using resource *k*, and *a*_*k*_ is the proportion of resources in the environment. In Sîrbu (2006), *a*_*k*_ represents the fraction of habitat category *k* within the total number of surveyed habitats.

In its original definition, *HS* was applied with categorical resources (e.g., habitat types, food items) or for gradients expressed as discrete series (e.g., time, season, counts of resource). For resources varying continuously along gradients (e.g., weight of food, caloric equivalent, tolerances to physical or chemical parameters of soil or water), the challenge of assessing the availability (the offer of the environment) and their use (requirements of the species) can be addressed with cubic spline interpolation functions and integral calculus (see the Online Resource). This is a novel methodological contribution proposed by us for ecological niches measured and compared along continuous resources.

*HS* serves as the basis for other measures and is applied in different methods.

We define the *standardized measure of niche dissimilarity* between species *i* and *j* as:

2 $${DHS}_{i,j}=1-{HS}_{i,j}$$

where *DHS*_*i,i*_ = *0*. *DHS* gives a symmetrical matrix of niche dissimilarities, having 0 on the main diagonal. We used this metric to further relate the sites-by-species values (C) to niche dissimilarities. For this purpose, we made a PCO analysis on the *DHS* matrix, saved the species scores on the coordinates axes (defining the N_all_ data table), and used an FWS procedure within a dc-CA for selecting those axes’ scores that best predicted the species composition while accounting also for their response to environmental variables. The result was the N matrix that entered the algorithm along with E, S, and T, for defining, measuring, and testing the CENT and CENTS spaces.

Another measure used as a synthetic parameter for community analysis was the mean amount of total difference between the niche of each species and all the others. We define *MSDHS*_*i*_ as the *mean niche difference or dissimilarity between species i and the other species within the community*, this being also a measure of the *species uniqueness* (sensu Ricotta et al. 2016):

3 $${MSDHS}_{i}=\frac{\sum_{j}{DHS}_{i,j}}{s}$$

For illustrative purposes, we used *MSDHS* as a trait, in a dc-CA, for linking communities to the environment.

We define the *ecological niche-based diversity measure FDen* as:

4 $${FDen}_{h}=\left(1/2\right)\sum_{i}\sum_{j}{DHS}_{i,j}{q}_{h,i}{q}_{h,j}$$

where *q*_*h,i*_ is the relative abundance of species *i* in the sample or site *h*. This is a modified variant of the Rao quadratic entropy measure (Rao 1982), which considers *DHS*. Because the product results in small values, and to standardize the range, we introduced a new variant (*FDsa*) and further used in our applications *the standardized ecological niche-based diversity measure FDsb* that ranges between 0 and 1:

5 $$FDs{a}_{h}=\left(1/2\right)\sum_{i}\sum_{j}\sqrt[3]{{DHS}_{i,j}{q}_{h,i}{q}_{h,j}}$$

6 $${FDsb}_{h}=\frac{\left[\left(1/2\right)\sum_{i}\sum_{j}\sqrt[3]{{DHS}_{i,j}{q}_{h,i}{q}_{h,j}}\right]-\mathrm{min}({FDsa}_{h})}{\mathrm{max}\left({FDsa}_{h}\right)-\mathrm{min}({FDsa}_{h})}$$

We compared the proposed ND indices with several measures of taxonomic diversity (TD): the number of species (*s*), Hill’s (N2) diversity measure (Hill 1973 ap. ter Braak and Šmilauer 2018), Shannon (H) entropy measure, and Pielou’s evenness index (J), which relates H to the logarithm of species number (Krebs 2014; ter Braak and Šmilauer 2018). To assess FD we used the standardized version (Šmilauer and Lepš 2014; ter Braak and Šmilauer 2018) of Rao functional diversity, but computed it only on those traits selected by forward selection (FWS) in the dc-CA. We performed a PCO on traits using Gower distance and then calculating Rao diversity (FD(Rao)) on those scores (PCOs) and the sites-by-species (C) table (Laliberté and Legendre 2010). We suggested and applied several methods to compare these measures and test their possible redundancy. We used our dataset for comparative purposes to show different ways to display these facets of diversity. We performed an RDA with all the described diversity metrics as response variables and E as predictors to plot and compare TD, FD, and ND. For this analysis, we constructed *t* value biplots (ter Braak and Šmilauer 2018) to evaluate the significance of the response of these diversity measures to each of the selected E. We performed a dc-CA with all the selected space and species predictors and plotted FD(Rao), FDen, and FDsb on the double-constrained space separately. We constructed a dendrogram based on the Pearson correlation coefficients using an average agglomerative method to compare all the diversity measures used in the analyses.

### Case study: freshwater mollusk communities from the middle Olt River

To illustrate the application of the measures and methods described here, we used our data on the habitats’ dimension of ecological niches (Sîrbu 2006) and the data from a study on freshwater mollusk community conducted in the middle Olt River basin, Romania. These data concern 23 species of freshwater mollusks from 20 sites. We conducted sampling and data analysis between March and July 2020. The area of reference is a river sector 83 km long, situated in the middle Olt River, which comprises a series of six reservoirs built in the second half of the twentieth century. The area extends from the town of Făgăraș (45.8512° N, 24.9733° E) to the Carpathian gorges (45.5317° N, 24.2721° E). The study aimed to explore responses of native communities to human pressure (hydro-technical works and others), and it will be published in extenso elsewhere.

For the dc-CA and other unimodal analyses, response data (weights in g) were log-transformed (by the relation *y*' = log(1 + *y*)) and standardized by sample total; thus, the final response variables were the relative dominance of species in terms of wet weight.

The selected E were flow (denoted Flow), an ordinal variable ranging from 1 (stagnant water, usually near the dams of the reservoirs) to 6 (rapid, turbulent flow in the gorges), an estimation of human impact (Impact), ranging from 1 (close to the natural condition of the river) to 6 (stagnant waters near the dams in the reservoir, with artificial banks and large parts of the substrate covered in concrete or other artificial materials), and the variable measuring the distance from the sampling station to the nearest dam downstream (Dis_dam, in m).

T were also selected by FWS in the dc-CA analysis. We selected maximal shell size of mollusks (SizeM), an ordinal variable ranging from (1) < 2.5 to (6) > 100 mm, and feeding type (FeedT), a factor with the levels: scraper (SCR), scraper and sediment feeder (SS), sediment feeder (SED), scraper and filter feeder (SF), filter feeder (F), sediment and filter, or suspension and deposit feeders (SEDF). The values for the traits were based on the works of Falkner et al. (2001), Glöer (2019), Killeen et al. (2004), Piechocki and Wawrzyniak-Wydrowska (2016), and Sîrbu and Benedek (2018).

To calculate the niche metrics, we used a dataset of species-by-habitat categories obtained during an earlier survey conducted between 1996 and 2003 (Sîrbu 2006). We used 28 types of habitats as resource descriptors. We considered the ratios of species occurrence in each of these categories as measures of resource use and the proportions of habitat categories within all the 405 surveyed habitats for assessing the resource availability in the research area.

We used PTC Mathcad 14 and PTC Mathcad Prime 5.0.0.0 (Parametric Technology Corporation) to calculate niche metrics and Canoco 5.12 (ter Braak and Šmilauer 2018) for the multivariate analyses and data visualization. We computed correlations between diversity indices using Hmisc package in R (Harrell 2020) and built the dendrogram in R version 3.6.1 (R Core Team 2019).

## Results

### CENT(S) variation partitioning for the mollusk dataset

**Fig. 2:**
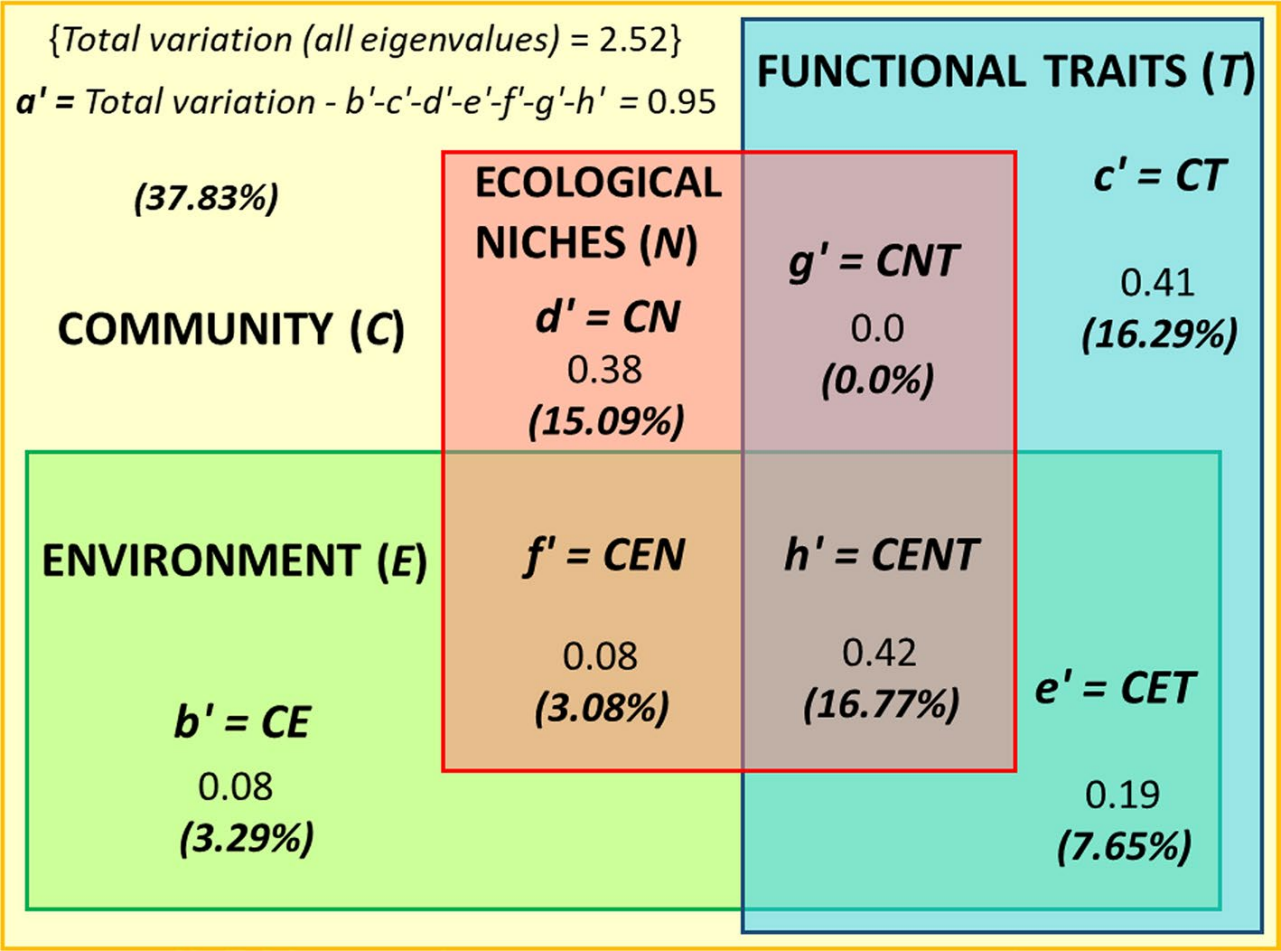
VADOC diagram showing decomposition of the variation in community composition explained by environment, traits, and niches. Letters *b*' to *h*' represent the parts of variation explained by different combinations of predictor groups, *h'* is the CENT (acronym from community–environment–niche–traits) overlap space, and *a'* represents the residual variation that the selected predictors cannot explain. The figures represent the components of variation in the mollusk communities in our case study, given both as the absolute variation explained and as percents of the total variation in community composition. 20 communities and 3 samples from each site (60 samples in total) were analyzed

**Fig. 3:**
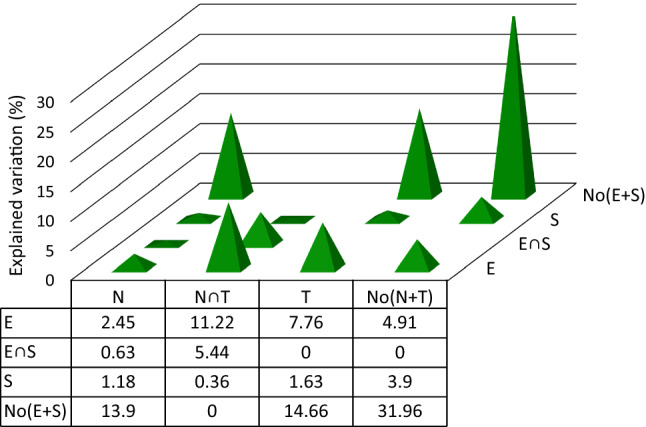
Another type of VADOC diagram, showing decomposition of the variation in community composition (C) explained by environment (E), space (S), traits (T), and niches (N). The figures represent the components of variation in the mollusk communities in our case study, given in percents of the total variation in community composition (all eigenvalues = total variation in *C* = 2.52). Predictors are identified by letters in caps, ∩ means the shared effects of two predictors, while No stands for none of the predictors included in parentheses. 20 communities and 3 samples from each site (60 samples in total) were analyzed

In our proposed algorithm for decomposition of the explained variation in C, the selection of dc-CA steps (Table 2 for CENT and Table 3 for CENTS analyses) depends on the questions asked in the particular study. In our case study, for illustration and discussion, we performed all the analyses and calculated all the variation parts (Fig. 2 for the CENT analysis, Fig. 3 for the CENTS analysis) and tested all the possible parts (or sums of parts) (Table 4). We present the VADOC diagrams in the two variants, the second (Fig. 3) being adapted based on a suggestion made by ter Braak (pers. comm.).

**Table 4:** Significance test results for the variation partitioning analyses

Step	dc-CA analysis	Significance tests (*p* all axes) from CCA	Significance test (p all) from dc-CA
I	CENT SPACE		
1.1'	C ~ (E) × (T|N)	{*b*' + *e*' + *f*' + *h*'} = 0.001 {*c*' + *e*'} = 0.058	{*e*'} = 0.004
1.2'	C ~ (E) × (N|T)	{*d*' + *f*'} = 0.061	{*f*'} = 0.382
1.3'	C ~ (E) × (T + N)	{*c*' + *d*' + *e*' + *f*' + *g*' + *h*'} = 0.005	{*e*' + *f*' + *h*'} = 0.001
1.4'	C ~ (E) × (T)	{*c*' + *e*' + *g*' + *h*'} = 0.024	{*e*' +* h*'} = 0.01
1.5'	C ~ (E) × (N)	{*d*' + *f*' + *g*' + *h*'} = 0.001	{*f*' + *h*'} = 0.001
II	CENTS SPACE		
1.1	C ~ (E|S) × (T|N)	{*b + e + h + f*} = 0.001 {*c + e + k + p*} = 0.058	{*e*} = 0.004
1.2	C ~ (E|S) × (N|T)	{*d + f + l + n*} = 0.061	{*f*} = 0.54
1.3	C ~ (E|S) × (T + N)	{*c + d + e + f + g + h + k + l + m + n + p + q*} = 0.005	{*e + f + h*} = 0.001
1.4	C ~ (E|S) × (T)	{*c + e + g + h + k + m + p + q*} = 0.024	{*e + h*} = 0.001
1.5	C ~ (E|S) × (N)	{*d + f + g + h + l + m + n + q*} = 0.001	{*f + h*} = 0.001
2.1	C ~ (S|E) × (T|N)	{*i + k + l + m*} = 0.088	{*k*} = 0.343
2.2	C ~ (S|E) × (N|T)		{*l*} = 0.271
2.3	C ~ (S|E) × (T + N)		{*k + l + m*} = 0.192
2.4	C ~ (S|E) × (T)		{*k + m*} = 0.231
2.5	C ~ (S|E) × (N)		{*l + m*} = 0.158
3.1	C ~ (E + S) × (T|N)		{*e + k + p*} = 0.002
3.2	C ~ (E + S) × (N|T)		{*f + l + n*} = 0.326
3.3	C ~ (E + S) × (T + N)	{*b + e + f + h + i + j + k + l + m + n + p + q*} = 0.001	{*e + f + h + k + l + m + n + p + q*} = 0.001
3.4	C ~ (E + S) × (T)		{*e + h + k + m + p + q*} = 0.001
3.5	C ~ (E + S) × (N)		{*f + h + l + m + n + q*} = 0.001
4.1	C ~ (E) × (T|N)	{*b + e + f + h + j + n + p + q*} = 0.001	{*e + p*} = 0.004
4.2	C ~ (E) × (N|T)		{*f + n*} = 0.385
4.3	C ~ (E) × (T + N)		{*e + f + h + n + p + q*} = 0.001
4.4	C ~ (E) × (T)		{*e + h + p + q*} = 0.001
4.5	C ~ (E) × (N)		{*f + h + n + q*} = 0.001
5.1	C ~ (S) × (T|N)	{*i + j + k + l + m + n + p + q*} = 0.036	{*k + p*} = 0.3
5.2	C ~ (S) × (N|T)		{*l + n*} = 0.141
5.3	C ~ (S) × (T + N)		{*k + l + m + n + p + q*} = 0.009
5.4	C ~ (S) × (T)		{*k + m + p + q*} = 0.014
5.5	C ~ (S) × (N)		{*l + m + n + q*} = 0.006

Codes of the explained variation parts are given in Fig. 2 for CENT and Fig. 3 for CENTS analyses

The total variation in the community composition was 2.52 (sum of all eigenvalues), all predictors explaining 68%. The remaining 32% was the residual variation (*a*), which cannot be explained by any of the selected predictors. Simple effects (variance in community composition explained by groups of predictors) were all significant (*p* on all axes < 0.05), similar and higher in T (explained variation is 40.5%) and N (34.8%), followed by E (30.8%) and S (11.5%). The strongest conditional (unique) effects were those of T and N (14.7% for T and 13.9% for N), compared to those of E (4.9%) and S (3.9%). Also, the lack of overlap between N and T in the absence of environmental and spatial predictors (their shared effect being null) clearly indicates that niche and traits, at least in our study, are anything but redundant, and adding niche data is beneficial for the model and for understanding the underlying mechanisms of communities structural and functional responses. In our study, if the niche data are removed, the unexplained variation in C rises from about one-third (32%) to about half (45.9%). This variation is also an argument in favor of using independent data on resource use of species in community ecology.

### Niche-based diversity indices

MSDHS, used as a functional trait in a dc-CA with E, explained 7.25% of the variation (6.58% adjusted) in the species–environment relationship, its effect being significant (*p* = 0.006 on all axes). This result shows that, if needed, some niche metrics can be used as a proxy for traits, in this case showing how distinctness of niches relates to environment predictors.

**Fig. 4:**
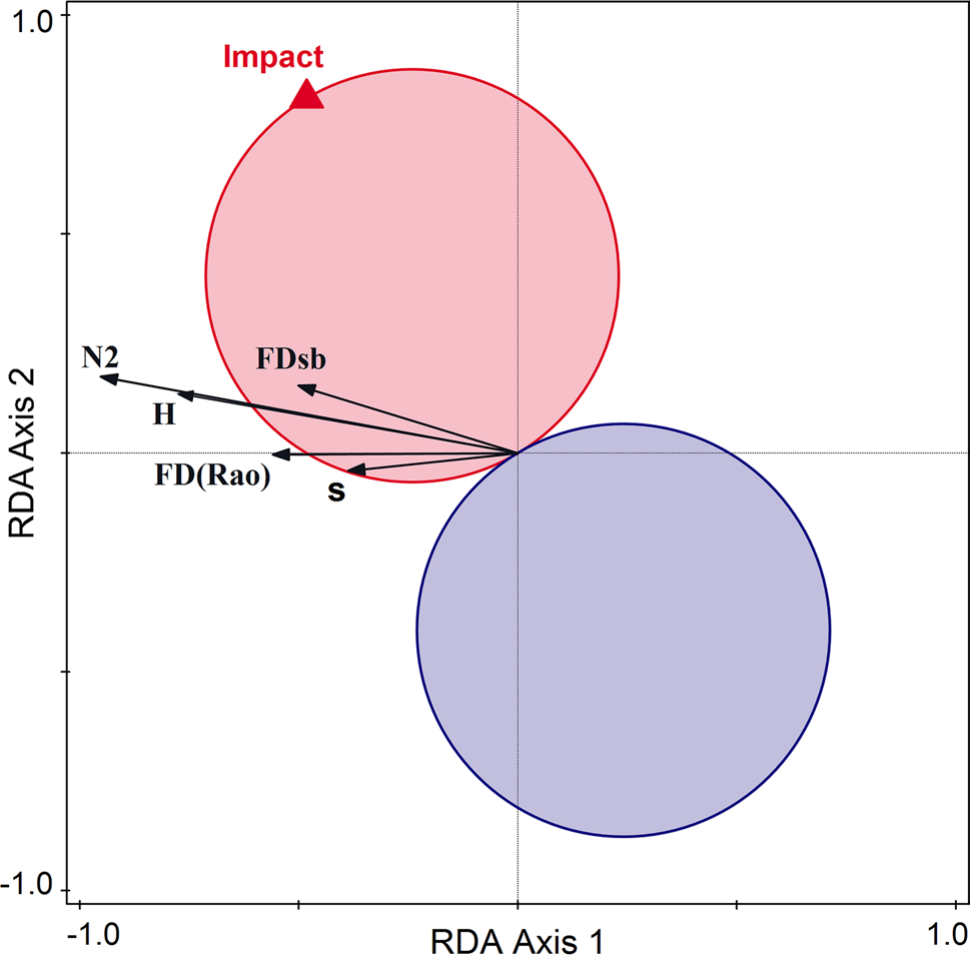
t-Value biplot (Van Dobben circles) showing the relationships between human pressures (Impact, as predictor) and the diversity measures (as response variables): number of species (s), N2—Hill's diversity measure, H—Shannon entropy measure, FD(Rao)—Rao's measure of functional diversity, FDsb—standardized niche-based diversity measure. The pink circle indicates the area of significant positive response of diversity measures to impact, and the blue circle the area of significant negative response. This is an attribute plot of the RDA between the diversity measures and the impact, distance to the dam and the flow as predictors; their simple effects are all significant, while the conditional effect is significant only for the Impact (*R*^2^ = 43.31%, P-adj = 0.006). 20 communities and 3 samples from each site (60 samples in total) were analyzed

In the RDA with TD, FD, and ND as response variables and E as predictors, the adjusted explained variation in the diversity measures was 34.4%, and the axes were significant (pseudo-F on all axes = 4.3, *p* = 0.013). The simple effect was significant for all three environmental variables, and it was strongest for Impact (43.3%, *p* = 0.006). However, conditional effects of Flow and the distance to the nearest downstream placed dam were not significant, showing their redundancy with the human pressure in explaining the mollusk diversity in our study area. FDsb and, marginally, *s* had a significant response (positive in this case) to Impact (Fig. 4).

**Fig. 5:**
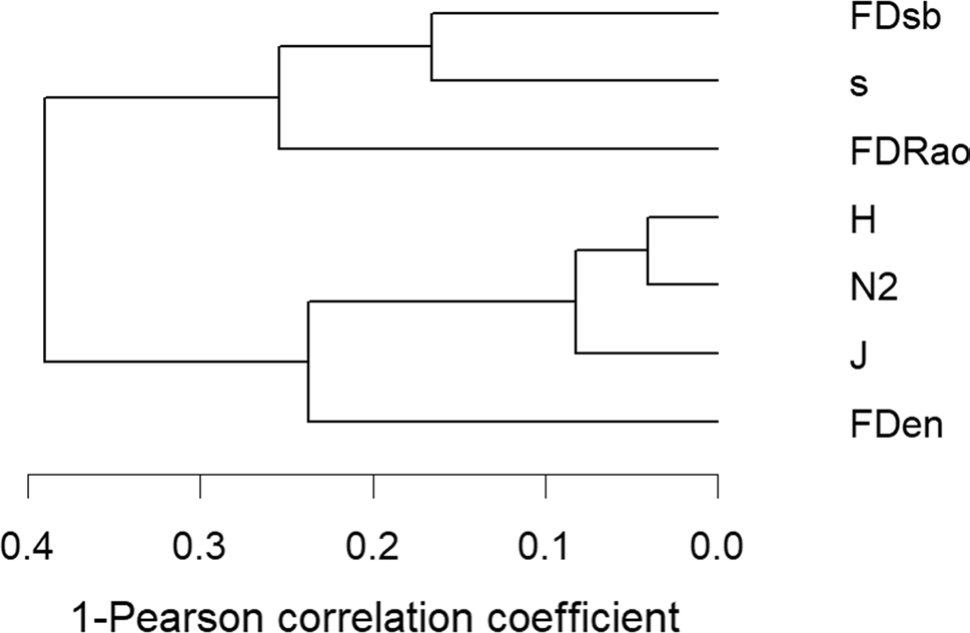
Dendrogram on the complement of Pearson correlation coefficients between diversity measures, built by the average agglomerative linkage method (diversity measures: FDsb standardized niche-based diversity, s number of species, FDRao Rao functional diversity, H Shannon entropy, N2 Hill's diversity, J Pielou's evenness index, FDen ecological niche-based diversity measure). 20 communities and 3 samples from each site (60 samples in total) were analyzed

In the dendrogram of diversity measures (Fig. 5), the two ND were not grouped, in contrast to the TD of heterogeneity (N2, H, and J). FDen joined at a greater distance the TD group, while FDsb was closely linked to *s*, and these two were further linked to FD(Rao). This way of illustrating relationships might help distinguish the meaning of the three different types of diversities (taxonomic, functional, and niche based) and also argues against their redundancy.

**Fig. 6:**
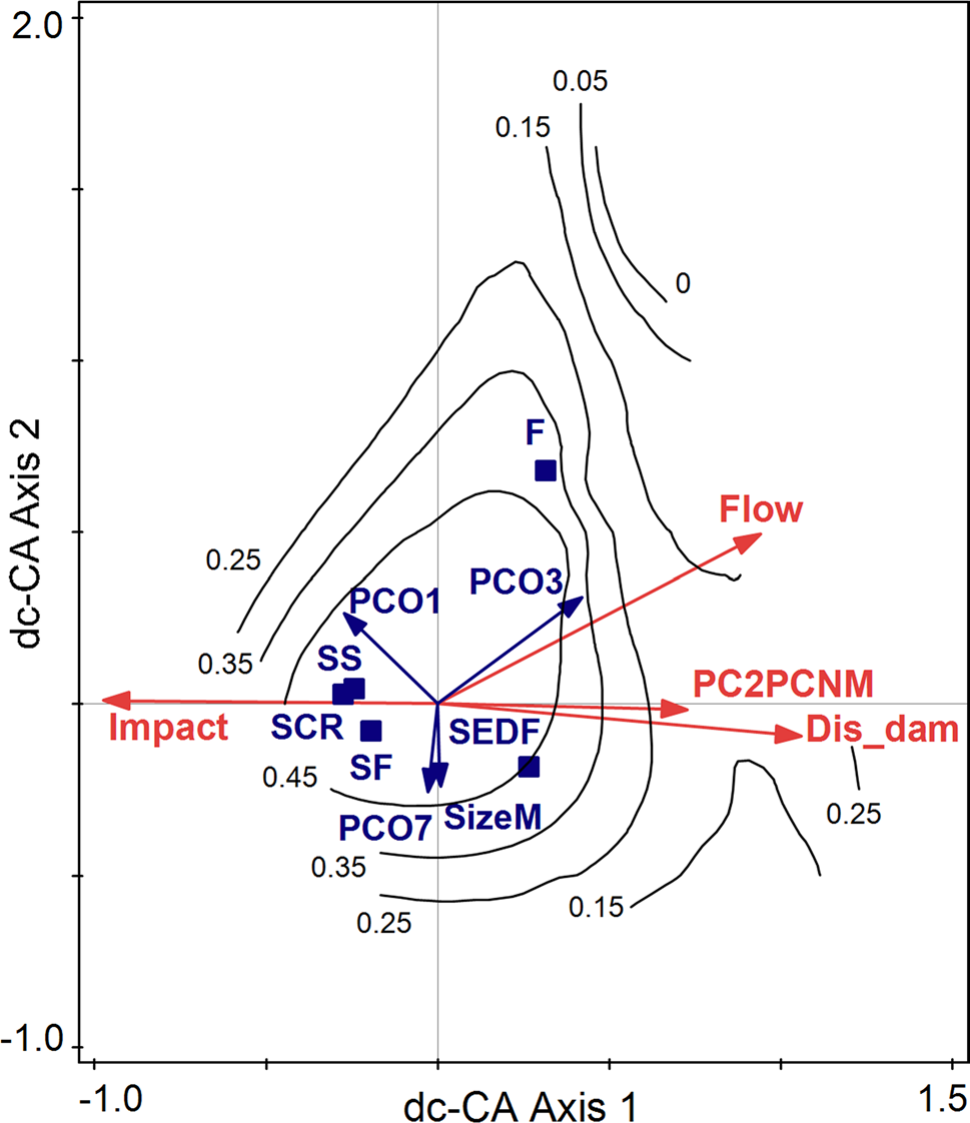
Double-constrained correspondence analysis (dc-CA) with all selected predictors and traits, with FDsb niche-based standardized diversity plotted by loess functions. *Impact* intensity of human impact, *Flow* flow, *Dis_dam* distance to the nearest dam downstream, *SizeM* maximal shell size of mollusks, *SCR* scraper, *SEDF* sediment and filter feeder, *SF* scraper and filter feeder, *SS* scraper and sediment feeder, *PC2PCNM* the second spatial eigenvector, selected from db-MEM,* PCO1, PCO3,* and *PCO7* first, third, and seventh PCOs' axes scores selected by forward selection from the axes resulting in principal coordinates analysis (PCO) on niche dissimilarities (the original DHS matrix). 20 communities and 3 samples from each site (60 samples in total) were analyzed

Another way to visualize the relationships of ND with the other variables is to build a dc-CA diagram with E, T, N, and S, and in that space to project one of these measures by either a loess function or by a GLM or GAM (the last two methods also allowing for testing the model significance), resulting in a contour plot with the isolines in the double-constrained space. We illustrated the variation of FDsb using a loess function (Fig. 6), the coefficient of determination being *R*^2^ = 51.8%. FDsb increased toward the average values of the environmental predictors (the intersection of axes) and decreased with Impact and Flow. By contrast, the FDen increased along the first double-constrained axis, showing a negative relationship with Impact, and the classical FD(Rao) showed a linear increase with the reduction of Flow and the increase of Impact (figures not shown here).

## Discussion

To expand the methodological framework of life–environment analyses, we modified the original three-table dc-CA method to introduce a five-table ordination algorithm (Fig. 1). Our approach combines, for the first time, the variability associated with the environment and locations where a species occurs and the variability associated with the traits and ecological niche of species within sites, incorporating niche predictors, separately and independently from traits. It also allows the decomposition of the variation in community composition, something that has been also done recently for the CEST space (Pinho et al. 2021). We show additional procedures for variation decomposition and testing in a five-matrix system, namely the CENTS space. In addition, various parts of the variation were tested and illustrated by a novel visualizing technique, the VADOC diagrams, which we present in two variants.

Our study reveals that evolutionary and adaptive signals may be of prevailing importance, compared to environmental and geographical predictors (the last categories being used more frequently), in explaining the quantitative structure of ecological communities. Using niche measures separately from traits might bring new insights into community assembly and reveal the adaptative features acquired by species during their evolution. Although usually used interchangeably, we argue that niche and traits descriptors can and should be treated as independent sources of information, and on a practical level, as different matrices in double-constrained analyses. Traits are characteristics identified mostly on the individual level, while niche metrics are features revealed on the species level, adding meaning and expanding the use of integrative methodology applied in ecology.

Peng et al. (2021) found dc-CA useful in assessing links between multiple stressors and ecosystem health, something we also emphasize by expanding its use and addressing the possibility to assess pressures, for instance, by measuring the significance and intensity of the relationship between the niche-based diversity and human impact.

Looking at spatial patterns may be important because the spatial distribution of species is directly influenced by environmental variables, while functional traits are expected to be indirectly related to space through their relationship with the environment (Pavoine et al. 2011). Therefore, if spatial signals are found in the traits but these are not correlated with the environment, some key environmental processes (present or past) might have been overlooked.

In their paper on trait–environment relationships of benthic macroinvertebrate communities sampled from the Danube River, Peng et al. (2021) chose not to separate the spatial component from the environmental one because, in their river dataset, most variables were highly correlated with the distance from the source, with the feeble possibility to separate their effects. In our study case, although the dataset also comes from sampling sites along a river, we chose to include the spatial predictors because the impact of anthropic activities, mainly dam building, alters the natural succession of environmental conditions along the river.

The environmental filtering hypothesis predicts that within a community, the environment selects species with similar traits (Grime 2006). Environmental filtering is thought to be a major mechanism structuring communities. However, our ability to accurately infer filtering based on community composition data was recently questioned by Tucker and Cadotte (2013), as competition can give rise to the same patterns as those caused by environmental filtering. Although distinguishing and testing the causes behind community assembly need manipulative studies of communities, including in the analyses of community structure, the ecological niche alongside species traits in correlation with the environment (and space) could help us understand the ecological and evolutionary processes that lead to certain species composition. In our approach to community assembly, we consider, and our study shows, that reducing ecological niches to an attribute characterized or derived from functional traits (Violle and Jiang 2009; Kearney et al. 2010) depletes the study of relationships between communities and their environment of an essential multivariate space of explanatory factors. Comparing the species through their niches may add new insight into evolutionary and adaptive constraints that link the structure and functions of ecological systems. Many studies have revealed that constrained analyses that relate groups of interacting populations to external factors (ter Braak and Šmilauer 2018; Šmilauer and Lepš 2014) need to be completed by adding information on space variability (Dray et al. 2006, 2012; Peres-Neto and Legendre 2010; Sharma et al. 2011), functional traits (Dolédec et al. 1996; Dray and Legendre 2008; Céréghino et al. 2018; ter Braak et al. 2018; Xu et al. 2019), and phylogenetic relationships (Desdevises et al. 2003; de Bello et al. 2005), to build and test a framework for understanding the underlying mechanisms of the responses of ecological systems to environmental changes, the laws that govern processes and offered services, and to predict their trajectories. We do not provide new methodology, but use existing knowledge and methods to step forward. Considering that traits and niche metrics characterize different ecological levels, introducing the niche in multivariate analyses aiming at linking communities to their environment means also testing for (dis)similarities between species concerning the use and partitioning of resources, adaptations, and interactions, i.e., for the intersection between their evolution and ecology.

If niche parameters are not available or desired, N can be a table of ecological indicator values for species, such as Ellenberg values in vascular plants, Grime’s CRS plant strategies, or saprobity values in diatoms (e.g., ter Braak 1987; ter Braak and van Dam 1989). This possibility extends the potential ecological area of application of the paper (ter Braak, pers. comm.).

The MSDHS characterizes the mean difference between the niche of a species and all the others. This niche-based index represents a novel measure of the species-facet level of redundancy (Ricotta et al. 2016) and can be used to evaluate derived measures, such as the *mean functional dissimilarity or vulnerability*. Further, MSDHS should be used for characterizing the community level of niche-based redundancy and the uniqueness of the functions associated with each species (*functional uniqueness*) by adapting the measures described by Ricotta et al. (2016). The derived measures should replace the *functional dissimilarities* with the *niche dissimilarities*, as described in Eqs. 4–6. MSDHS may also enter new analyses (of multiple regression, for instance) as a synthetic response variable or, as shown before, to be considered as a trait within a dc-CA. De Cáceres et al. (2011) provide a framework for calculating resource niche metrics (space resource) using the distance-based PCO approach. Instead, we have chosen a direct ordination method, but all the metrics introduced by De Cáceres et al. (2011) can be easily calculated on the indices and used in the methods we have presented here.

Assessing FD in ecological communities is very promising for studying the response of diversity to environmental gradients and the effects of diversity on ecosystem functioning (Lepš et al. 2006) as well as on ecosystem functions and services (de Bello et al. 2010, 2016; Cadotte et al. 2011; Boersma et al. 2016; Carmona et al. 2016). Defining and measuring relationships between the different types of diversity is a complex task (Mouchet et al. 2010; de Bello et al. 2010, 2016; Pavoine and Bonsall 2011; Lepš 2013; Carmona et al. 2016). We have shown that both FDen and FDsb (as measures of ND) are not redundant with other diversity measures, including FD(Rao), but explain in a complementary manner the responses of communities to environmental gradients. Thus, they should not be used as a proxy for each other, at least not a priori. In our case study, FDsb proved to be more sensitive to human impact than other diversity measures, suggesting that ND could be a valuable tool in environmental monitoring and ecological management and as an indicator when evaluating success in conservation projects.

There is a growing interest in using FD for developing new methods for environmental monitoring and human impact evaluation and mitigation (Cianciaruso et al. 2009; Laliberté and Legendre 2010; Cadotte et al. 2011; Boersma et al. 2016) and for life cycle assessment (Ahmed et al. 2018). We agree with all these and add that ND measures should be used in the future, since they show the same possibilities and are not redundant with the classical FD measures. Although relying on (and inspired by) the Rao entropy measure, their levels of reference and meaning are different.

The methods presented here are useful when applied to tables with high variability of data (biological and environmental). For this reason, we selected for illustrative purposes data from a heterogeneous river sector, where the structure of the mollusk communities reflects the diversity of habitats (ranging from flowing to stagnant waters in the reservoirs), environmental conditions, and sources of human impact. The study group is also heterogeneous, including gastropods and bivalves of different origins (natives, native invasive, allochthonous invasive) with various traits.

### Expanding further the framework

As Pavoine et al. (2011) suggested for their method using RLQ, our algorithm can also be extended to address other key issues in ecology, such as the interspecific relationships between plants and herbivores or pollinators. In this case, the traits and niche of herbivores or pollinators will be related to the traits and niche of plants, with the possibility of accounting for the direct effect of environment and space on plants and their indirect effect on herbivores.

Phylogeny is considered another source of variation and explanation in the relationship between species and environment regardless of whether traits are included or not in the analysis (Desdevises et al. 2003; Šmilauerova and Šmilauer 2007; Pavoine and Bonsall 2011; de Bello et al. 2017; Xu et al. 2019). Our integrative approach could be further expanded, including the sixth matrix of patristic distances or genetic distances, depending on the available data and research questions. This expansion could be done by combining our algorithm with that proposed by Desdevises et al. (2003) and using specific tests and related methods (Campbell et al. 2011).

In effect, the same principles and methods of variation partitioning of two predictor tables might be used for three or more tables. Our 2 × 2 variation partitioning algorithm in dc-CA can be extended to a 3 × 3 algorithm by adding, for instance, invasive species as site descriptors and phylogeny as species descriptors. Further, this extension could be generalized into an (*n* × *k*) variation partitioning, where *n* tables are related to the sites and *k* to the species. Besides, our illustration of results may be used directly to interpret both simple and conditional effects of various kinds of predictors on the community structure, relying on the ability to test the fractions of explained variance. Besides, if a non-trivial experimental design is involved, the permutation scheme in dc-CA can be easily adapted to suit and reflect the sampling protocol.

In dc-CA, the number of both environmental variables (space predictors included) and traits (niche predictors included) needs to be significantly smaller than the number of cases and species (ter Braak and Šmilauer 2018). Therefore, our algorithm is not suitable for datasets with many predictors and few cases (sites and species). An alternative may be to replace the dc-CA with a double-asymmetrical co-correspondence analysis (CoCA, which considers unimodal responses to underlying gradients) or its linear counterpart, the co-inertia analysis (CoIA), which can be used with many predictors and few objects (cases or samples). Although non-symmetrical CoCA can be done by existing R packages and functions, the double-asymmetrical CoCA and CoIA still have to be developed.

Another possible expansion is to step outside the two-dimensional table of the double-constrained method and adopt a multi-level approach, such as an *n*-dimensional constrained analysis. By this expansion, we propose the theoretical and methodological development toward an ‘omni-spaces explanatory ecology of communities’: by this, meaning the consideration and use of an unlimited number and types of explanatory data tables, constraining either sites or species (rows and columns), in any number of response data sets (multiple interacting or linked communities).

Unlike environmental variables and traits, which have a straightforward meaning, the significance and underlying mechanisms of niche overlap may vary greatly, depending on how the niche is defined and what data are used to evaluate it. Here, we have illustrated a relatively simple case study where we included the use and partitioning of habitat types, related mainly to life–environment interactions. When food resources or interspecific relationships are considered, the focus will be on biotic processes. In complex studies, considering different aspects of the niche, several niche overlap matrices can be combined, calculating the centroids, the mean, or another measure of central tendency of the similarities for each pair of species.

## Conclusions

Including ecological niche metrics, especially niche dissimilarities, in the CENTS model, as a separate data table, adds the functional interaction between the species to the model that accounts for distribution, space variability, environmental predictors, and biological traits, and sometimes also for phylogenetic relationships. This can lead to new theoretical and applied research objectives. We have shown that one can use an existing three-table ordination method to analyze relationships among five or more datasets. Maybe the future will show that for challenging the complex environmental issues related to overpopulation, resource exploitation, degradation of habitats, invasive species, pollution, and loss of ecological functions, new methods have to be developed for linking more datasets from different informational spaces. The multivariate approach is already mature; new needs arise by linking and integrating multi-spaces, each of them being a system of multivariate datasets linked within and among the spaces. Searching for solutions to such complex issues will no longer result in predicting a structure or a group of functions, but tracing and modeling complex trajectories of ecological systems simultaneously along time, space, and other gradients. This will also mean a complex and multidisciplinary approach and the development of new mathematical tools for assisting the needs of ecological research. In this frame, we completed only a small step but showed the urge to move forward.

## Supplementary Information

Below is the link to the electronic supplementary material.

Supplementary file1 (DOCX 238 KB)

## Data Availability

The datasets used and/or analyzed during the current study are available from the corresponding author on reasonable request and will be soon available on a digital repository.

## Code availability

The statistical software R is freeware and Canoco is licensed, and its license is the personal property of the corresponding author. Mathcad is licensed, and we used a Mathcad Education – University Edition Subscription license in 2020.
