# Supplementary material for: Variation partitioning in double-constrained multivariate analyses: linking communities, environment, space, functional traits, and ecological niches
Source: Oecologia. 2021 Aug 11;197(1):43–59. doi: 10.1007/s00442-021-05006-6 (PMC8445867; doi:10.1007/s00442-021-05006-6)
Supplement: Supplementary file 1 — Supplementary file1 (DOCX 238 KB) [file 442_2021_5006_MOESM1_ESM.docx]

**Variation partitioning in double-constrained multivariate analyses: linking communities, environment, space, functional traits, and ecological niches**

DOI: 10.1007/s00442-021-05006-6

Ioan Sîrbu, Ana Maria Benedek, Monica Sîrbu

Correspondence author: Ioan Sîrbu

Lucian Blaga University of Sibiu, Faculty of Sciences,

5-7 Dr. I. Ratiu St., 550012 Sibiu, Romania

[ioan.n.sirbu@ulbsibiu.ro](mailto:ioan.n.sirbu@ulbsibiu.ro), [meosirbu@yahoo.com](mailto:meosirbu@yahoo.com)

**Online Resources**

**1. Algorithm for variation partitioning of Community – Environment – Niche – Trait (CENT) and Community – Environment – Niche – Trait – Space (CENTS) spaces without using covariates**

The decomposition of the community space using double constrained correspondence analysis (dc-CA) is based on the idea that the canonical (double-constrained) eigenvalues resulting from this analysis represent the intersection between the effects of the environment and traits on species composition. In the variation partitioning, the explained variation is decomposed in the two conditional (unique) effects of the two predictors (or, more often, groups of predictors) and their overlap. Using a combination of canonical correspondence analyses (CCAs), or their linear counterparts redundancy analyses (RDAs), and dc-CAs or their linear counterparts double constrained principal component analyses (dc-PCAs), without covariates, one can calculate the CENT or CENTS variation parts (Table 1) but may test only the simple effects. The significance for each of the simple effects is given by the (double) constrained test on all ordination axes performed during the analyses. When only one dominant gradient is expected in the data, the significance of the test on the first axis will be considered.

Table 1 Codes for Community (C) – Environment (E) – Niche (N) – Trait (T) for the (CENT) and Community – Environment – Niche – Trait – Space (S) for the (CENTS) variation parts

|  | unique effect of T | unique effect of N | shared effect of  T and N | unexplained by  T and N |
| --- | --- | --- | --- | --- |
| CENT | | | | |
| effect of E | e' | f' | h' | b' |
| unexplained by E | c' | d' | g' | a' |
| CENTS | | | | |
| unique effect of E | e | f | h | b |
| unique effect of S | k | l | m | i |
| shared effect of E and S | p | n | q | j |
| unexplained by E and S | c | d | g | a |

The algorithm for the decomposition of CENT space is explicitly given in Table 2, and the algorithm for CENTS is synthesized in Table 3. In both algorithms, step 0 is represented by the unconstrained correspondence analysis (CA) of the community (C), which returns the total variation in the response variables table, meaning the total eigenvalues.

The algorithm for decomposition of the CENT space (Table 2) comprises two steps, in addition to step 0. The first step has four substeps (1.1' to 1.4') represented by the CCAs measuring and testing the individual effect of environment (E), traits (T), niche (N), and the combined effect of the last two (T+N) on community (C). The second step has three substeps (2.1' to 2.3') represented by the dc-CAs in which the environmentally structured variation of C is related to T+N, T, and respectively N.

Table 2 Algorithm for variation partitioning of CENT space relating communities (C) to the environment (E), traits (T), and niche (N). The tilde ~ stands for canonical ordination analysis, followed by the predictors placed in round brackets, separated by the × symbol, for discriminating between predictors related to sites (E) and those linked to species (T or N or both). The total variation (all eigenvalues, coded as All_eg) is given by the correspondence analysis of C (CA C). Canonical eigenvalues are coded as ceg, and the predictors used are enclosed in brackets []. The lowercase letters correspond to the variation parts given in Table 1. Significance tests are related to the variation parts given in braces {}, also using the formerly mentioned letters.

| Step | Analysis | Estimates | Significance tests (p all axes) |
| --- | --- | --- | --- |
| 0 | CA C | Total variation = All_eg |  |
| 1.1' | CCA C~(E) | ceg[E] | {b'+e'+f'+h'} |
| 1.2' | CCA C'~(T) | ceg[T] | {c'+e'+g'+h'} |
| 1.3' | CCA C'~(N) | ceg[N] | {d'+f'+g'+h'} |
| 1.4' | CCA C'~(T+N) | ceg[T+N] | {c'+d'+e'+f'+g'+h'} |
| 2.1' | dc-CA C~(E)×(T+N) | b'=ceg[E]-ceg[(E)×(T+N)] | {e'+f'+h'} |
| 2.2' | dc-CA C~(E)×(T) | f'=ceg[(E)×(T+N)]-ceg[(E)×(T)] | {e'+h'} |
| 2.3' | dc-CA C~(E)×(N) | e'=ceg[(E)×(T+N)]-ceg[(E)×(N)]  h'=ceg[(E)x(T)]-e'  d'=ceg[T+N]-ceg[T]-f'  c'=ceg[T+N]-ceg[N]-e'  g'=ceg[T]-c'-e'-h'  a'=All_eg-b'-c'-d'-e'-f'-g'-h' | {f'+h'} |

The algorithm for decomposition of the CENTS space (Table 3) comprises four steps, in addition to step 0. The first step has six substeps (1.1 to 1.6) represented by the CCAs measuring and testing the combined effects of E+S and T+N and their individual effects on C. Each of the following three steps has three substeps represented by the dc-CAs. In steps 2.1 to 2.3, the environmentally and spatially structured variation of C is related to the combined effects of T+N and their individual effects. In steps 3.1 to 3.3, the environmentally structured variation of C is related to the combined effects of T+N and their individual effects. In steps 4.1 to 4.3, the spatially structured variation of C is related to the combined effects of T+N and their individual effects.

Table 3 Algorithm for variation partitioning of CENTS space relating communities (C) to the environment (E), space (S), traits (T), and niche (N). The tilde ~ stands for canonical ordination analysis, followed by the predictors placed in round brackets, separated by the × symbol, for discriminating between predictors related to sites (E or S or both) and those linked to species (T or N or both). The total variation (all eigenvalues, coded as All-eg) is given by the correspondence analysis of C (CA C). Canonical eigenvalues are coded as ceg, and the predictors used are enclosed in brackets []. The lowercase letters correspond to the variation parts given in Table 1. Significance tests are related to the variation parts given in braces {}, also using the formerly mentioned letters.

| Step | Analysis | Estimates | Significance tests (p) |
| --- | --- | --- | --- |
| 0 | CA C | Total variation = All_eg |  |
| 1.1 | CCA C~E+S | ceg[E+S] | {b+e+f+h+i+j+k+l+m+n+p+q} |
| 1.2 | CCA C'~T+N | ceg[T+N] | {c+d+e+f+g+h+k+l+m+n+p+q} |
| 1.3 | CCA C~E | ceg[E] | {b+e+f+h+j+n+p+q} |
| 1.4 | CCA C~S | ceg[S] | {i+j+k+l+m+n+p+q} |
| 1.5 | CCA C'~T | ceg[T] | {c+e+g+h+k+m+p+q} |
| 1.6 | CCA C'~N | ceg[N] | {d+f+g+h+l+m+n+q} |
| 2.1 | dc-CA C~(E+S)×(T+N) | ceg[(E+S)x(T+N)] | {e+f+h+k+l+m+n+p+q} |
| 2.2 | dc-CA C~(E+S)×(T) | d=ceg[T+N]-ceg[(E+S)×(T+N)]-ceg[T]+ceg[(E+S)×(T)] | {e+h+k+m+p+q} |
| 2.3 | dc-CA C~(E+S)×(N) | c=ceg[T+N]-ceg[(E+S)×(T+N)]-ceg[N]+ceg[(E+S)×(N)]  g=ceg[T]-ceg[(E+S)×(T)]-c | {f+h+l+m+n+q} |
| 3.1 | dc-CA C~(E)×(T+N) | i=ceg[E+S]-ceg[(E+S)×(T+N)]-ceg[E]+ceg[(E)×(T+N)] | {e+f+h+n+p+q} |
| 3.2 | dc-CA C~(E)×(T) | l=ceg[(E+S)×(T+N)]-ceg[(E+S)×(T)]-ceg[(E)×(T+N)]+ceg[(E)×(T)] | {e+h+p+q} |
| 3.3 | dc-CA C~(E)×(N) | k=ceg[(E+S)×(T+N)]-ceg[(E+S)×(N)]-ceg[(E)×(T+N)]+ceg[(E)×(N)] | {f+h+n+q} |
| 4.1 | dc-CA C~(S)×(T+N) | b=ceg[E+S]-ceg[(E+S)×(T+N)]-ceg[S]+ceg[(S)×(T+N)]  j= ceg[E]+ceg[(E)×(T+N)]-b | {k+l+m+n+p+q} |
| 4.2 | dc-CA C~(S)×(T) | f=ceg[(E+S)×(T+N)]-ceg[(E+S)×(T)]-ceg[(S)×(T+N)]+ceg[(S)×(T)]  n= ceg[(E+S)×(T+N)]-ceg[(E+S)×(T)]-f-l | {k+m+p+q} |
| 4.3 | dc-CA C~(S)×(N) | e=ceg[(E+S)×(T+N)]-ceg[(E+S)×(N)]-ceg[(S)×(T+N)]+ceg[(S)×(N)]  p=ceg[(E+S)×(T+N)]-ceg[(E+S)×(N)]-e-k  h=ceg[(E+S)×(T+N)]-ceg[(S)×(T+N)]-e-f  m=ceg[(E+S)×(T+N)]-ceg[(E)×(T+N)]-k-l  q=ceg[(S)×(N)]-l-m-n  a=All_eig-b-c-d-e-f-g-h-i-j-k-  -l-m-n-p-q | {l+m+n+q} |

The graphical display of these results can be done by VADOC diagrams (term derived from 'variation partitioning in double-constrained ordination analyses with multiple predictor tables diagram'), as shown in the article main text, or included in a table format (Fig. 1).


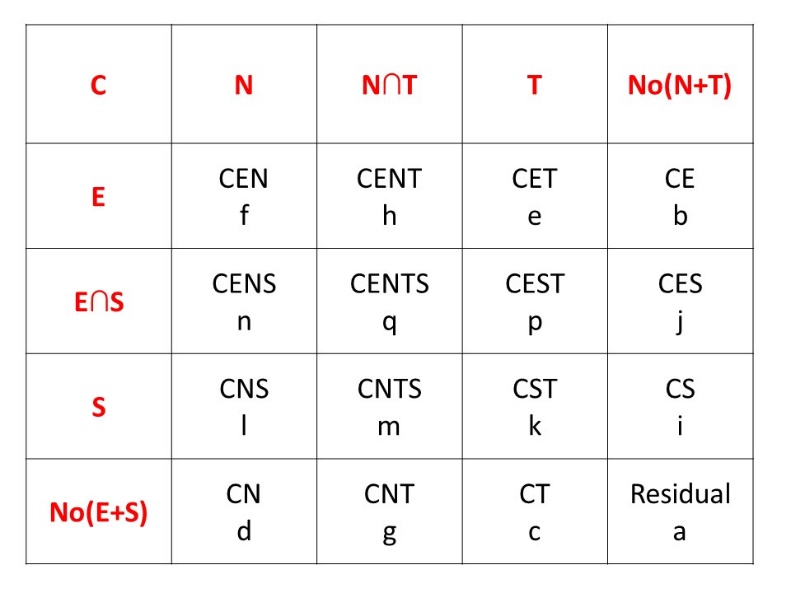


**Fig. 1** Possible display of the variation partitioning results. Capital letters are the selected variables standing for effects of N = ecological niche, T = functional traits, E = environmental variables, S = space predictors. No(N+T) and No(E+S) indicate the variation part that is not explained by the predictors in the parentheses. The symbol ∩ stands for the intersection (i.e., the overlap between N and T, and between E and S). C is the ecological community, and its residual variation (Residual a) is the part of the total variation (sum of all eigenvalues) that is not explained by any predictor or combination

**2. Algorithm for variation partitioning of Community – Environment – Space – Trait (CEST) space**

In some studies, ecological niche data is not available or is of no research interest. Therefore we provide here two algorithms for the decomposition of CEST space (Fig. 2 and Fig. 3) and testing the effects of the predictor groups. The algorithms are based on a combination of CCAs and dc-CAs. The significance of the simple and conditional effects is given by the (double) constrained test on all ordination axes performed during the analyses. When only one dominant gradient is expected in the data, the significance of the test on the first axis will be considered. In both algorithms, step 0 is represented by the unconstrained CA of C, which returns the total variation in the response variables table, meaning the total eigenvalues. The unadjusted percentages of explained variation can be calculated by dividing the various variation parts by the total variation in the community composition, extracted from the species-by-sites table in the CA.


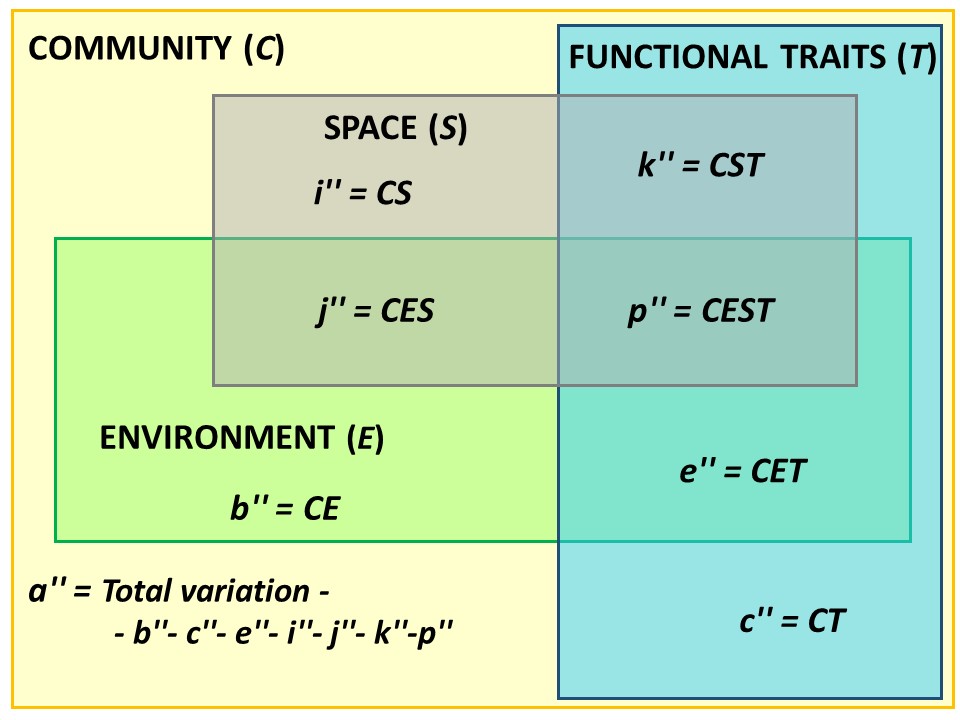


**Fig. 2** VADOC diagram (variation partitioning in double-constrained ordination analyses with multiple predictor tables diagram) illustrating the decomposition of CEST space

The first algorithm (Table 4) is adapted for Canoco 5.1, which allows the use of covariates in double constrained analyses and also provides the results of the intermediate steps, i.e., the two CCAs testing the constraints either on sites, with (E) or (S) or both as predictors, or on species, with (T) or (N) or both as predictors. This algorithm with covariates allows the easy calculation of CEST variation parts and testing both simple and conditional effects.

The second algorithm (Table 5) is adapted for other software (such as R), which does not allow (yet) the use of covariates in dc-CA and does not provide the intermediate results (of the CCAs). This algorithm without covariates allows only the testing of simple effects.

The results of the variation decomposition of CEST space in our study case are given in Fig. 3, showing another variant of VADOC diagram (suggested by Ter Braak, pers. comm.).

Table 4 Algorithm for variation partitioning of CEST space with covariates, relating communities (C) to the environment (E), space (S), and traits (T). The tilde ~ stands for canonical ordination analysis, followed by the predictors placed in round brackets, separated by the × symbol, for discriminating between predictors related to sites (E or S or both) and those linked to species (T). The total variation (all eigenvalues, coded as All_eg) is given by the correspondence analysis of C (CA C). Canonical eigenvalues are coded as ceg, and the predictors used are enclosed in brackets []. Vertical bar | separates the covariates (to the right of it), the lowercase letters correspond to the variation parts given in Fig. 2. Significance tests are related to the variation parts given in braces {} for the CCA and {}^dc^ (with superscript) for the dc-CA, also using the formerly mentioned letters.

| Step | Analysis | Estimates | Significance tests (p) |
| --- | --- | --- | --- |
| 0 | CA C | Total variation = All_eg |  |
| 1.1 | dc-CA C~(E\|S)×(T) | e''=ceg[(E\|S)×(T)]  b''= ceg[E\|S]-e'' | {c''+e''+k''+p''}  {b''+e''}  {e''}^dc^ |
| 1.2 | dc-CA C~(S\|E)×(T) | k''=ceg[(S\|E)×(T)]  i''=ceg[S\|E]-k'' | {i''+k''}  {k''}^dc^ |
| 1.3 | dc-CA C~(E+S)×(T) | p''=ceg[(E+S)×(T)]-e''-k''  c''=ceg[T]-ceg[(E+S)×(T)] | {b''+e''+k''+i''+j''+p''}  {e''+k''+p''}^dc^ |
| 1.4 | dc-CA C~(E)×(T) | j''=ceg[E]-b''-e''-p''  a''=All_eg-b''-c''-e''-k''-i''-j''-p'' | {b''+e''+j''+p''}  {e''+p''}^dc^ |
| 1.5 | dc-CA C~(S)×(T) |  | {k''+i''+j''+p''}  {k''+p''}^dc^ |

**Fig. 3** Variation decomposition of CEST space (VADOC diagram); case study: the mollusk communities from the Olt River. E = unique effect of environment predictors, S = unique effect of space predictors, E∩S = shared effect of environment and space, No(E+S) = variation in community composition not explained by environment and space predictors, T = effect of species traits, No(T) = variation in community composition not explained by species traits. Percents of explained variation are not adjusted (explained variation given as percentage of the total variation). 20 communities and 3 samples per site (60 samples in total) were analyzed.

Table 5 Algorithm for variation partitioning of CEST space without covariates, relating communities (C) to the environment (E), space (S), and traits (T). The tilde ~ stands for canonical ordination analysis, followed by the predictors placed in round brackets, separated by the × symbol, for discriminating between predictors related to sites (E or S or both) and those linked to species (T). The total variation (all eigenvalues, coded as All_eg) is given by the correspondence analysis of C (CA C). Canonical eigenvalues are coded as ceg, and the predictors used are enclosed in brackets []. The lowercase letters correspond to the variation parts given in Fig. 2. Significance tests are related to the variation parts given in braces {}, also using the formerly mentioned letters.

| Step | Analysis | Estimates | Significance tests (p) |
| --- | --- | --- | --- |
| 0 | CA C | Total variation = All_eg |  |
| 1.1 | CCA C'~(E) |  | {b''+e''+j''+p''} |
| 1.2 | CCA C'~(S) |  | {i''+k''+j''+p''} |
| 1.3 | CCA C'~(E+S) |  | {b''+i''+e''+k''+j''+p''} |
| 1.4 | CCA C~(T) |  | {c''+e''+k''+p''} |
| 2.1 | dc-CA C~(E+S)×(T) | c''=ceg[T]-ceg[(E+S)×(T)] | {e''+k''+p''}^dc^ |
| 2.2 | dc-CA C~(E)×(T) | k''=ceg[(E+S)×(T)]-ceg[(E)×(T)] | {e''+p''}^dc^ |
| 2.3 | dc-CA C~(S)×(T) | e''=ceg[(E+S)×(T)]-ceg[(S)×(T)]  p''=ceg[T]-c''-e''-k''  i''=ceg[E+S]-ceg[E]-k''  b''=ceg[E+S]-ceg[S]-e''  j''=ceg[E]-b''-e''-p''  a''=All_eg-b''-c''-e''-k''-i''-j''-p'' | {k''+p''}^dc^ |

**3. Computing HS niche similarity measure for continuous resources**

For resources varying continuously along gradients (e.g., weight of food, caloric equivalent, tolerances to physical, or chemical parameters of soil or water), the challenge of assessing the availability (the offer of the environment) and the use (requirements of the species) can be addressed with cubic spline interpolation functions and integral calculus. The proposed algorithm for this method assumes that the measurements are done along continuous gradients of both environmental descriptors and resources related to the gradients, but the measurements are done at predefined, fixed, or selected values of the gradient. In each of the selected coordinates of the environmental gradient, which also serves as the abscissa, the values of resources in the environment and their use by the species are arranged in corresponding vectors, and these will serve as the basis for ordinates. Cubic spline curves on segments can be used for defining the interpolation functions, which model the variation of the dependent variables (resources status and use) against the gradient. The surface between the function and the abscissa is estimated by the integral, defined on the range (or segments) of the gradient, and this gives an estimation of the total resources use and the resource abundance along that sector.

If the resource availability (its abundance, weight, or others, using either instantaneous or accumulative measures) is also assessed during the field work or from databases (e.g., statistics like central limits, variances, or sums of hydrological or climatic parameters) and its variation along the given gradient is known, the method described before will conclude in the evaluation of its status in the environment. Thus, the results for each species and resource will be an area evaluated by the integral of its spline function, or several values if segments of the gradient are defined. The resource's availability can be assessed in two variants, depending on the existence of values of reference (e.g., maximal, obtained in areas or years of optimum values of the environment, or compared to optimal values when no limiting factors are acting) or, in the absence of these, as a ratio of the sum of all available resources. If there are no reference or optimal values, then the interpolation function of *r* ratios to the total amount (∑*r*) is plotted, and the area is assessed. If values of reference *R_k_* are available, then ratios between *r_k_/R_k_* are first computed, and these will be treated in the same way as described before. Thus, the availability *a_i_* of any resource is either *r_i_/R_i_* (expressed as the proportion of the total sum) or *r_i_/∑r_i_*  that can be further used as the product term *p_i_a_i_* in the measures (niche metrics defined in the main text, equations 1 to 6). If partial cumulative ratios are necessary for the research (sums of *r* or *r/R* for the first *j* terms*,* where *1<j<k),* then a row of integrals defined on each subsector of the gradient will be used.

The described method is detailed on an imaginary dataset, using a Mathcad 14 template. The whole methodological algorithm is given, and each step is explained and can be therefore used as such in the specific software. The reason for using cubic spline curves (or interpolation functions) is the low variation in the curves' shape that mimic natural fluctuations, and model more naturally the variation of ecological and environmental processes. Any other interpolation method and functions could be used instead, if appropriate.

If *x* is the gradient (varying between min(*x*) and max(*x*)), *z* is an index of summation, serving as a proxy for continuous increase in *x*, for instance, beginning with min(x) and an added lag of 0.1 (smaller or larger, depending on the research conditions), the interpolation function for a species 1 that uses or exploits a certain resource is *fsp1(z)*, and for the species 2 is *fsp2(z)*, while the interpolation function for the resource status or ratios in the environment (with or without values of reference or for optimality R), defined as *gsa(z)*, then the HS measure of niche similarity or overlap between two species, becomes:

${HS}_{12}=\frac{\int_{min(x)}^{max(x)} gsa(z)\sqrt{fsp1\left( z \right)fsp2(z)}dz}{\sqrt{\left( \int_{min(x)}^{max(x)} gsa\left( z \right)fsp1\left( z \right)dz \right)\left( \int_{min(x)}^{max(x)} gsa\left( z \right)fsp2\left( z \right)dz \right)}}$ (7)

The same equation is valid for each i and j pair of species, resulting in a *s* x *s* matrix of niche measures of similarities (*s* = number of species). HS on continuous resources also varies between 0 and 1.

If the resources' use and status are measured by continuous methods, and the functions are already known or obtained by mathematical modeling, then interpolation is no more of use, and only the integrals will be used for the measures. Depending on the data and hypotheses, an alternative is using regression models (GLM, GAM) instead of interpolation functions.

When a range of measurements are available (e.g., *i* values of a given chemical parameter), for each value *c*_i_ measured in a site, the standardized position of this measurement could be expressed as *r_i_=(c_i_ - C_min_)/(C_max_-C_min_*), where the range is the difference between the maximum value *C_max_* and the minimum *C_min_*. In this variant, equations are used as described for categorical resources.

The Mathcad template for evaluating the HS standardized measure of ecological niches overlap or (dis)similarities for continuous resources is given below, where:

x = the gradient along which the resources (their availability and consumption or use) are measured. It can be the altitude, a chemical concentration range, distance from a fixed point or impact source, or others.

R = the vector with values of reference for the resources, i.e., values in the whole area, multiannual values, or maximal possible values, showing, for instance, the ideal status of resource when the limiting factors or adverse forces are removed or mitigated.

r = resource's availability, i.e., real or realized value of the particular resource in the environment, during the research timespan corresponding to the evaluation or to a given moment.

Sp1, Sp2 = resource use of two species in 10 sites placed along a continuous gradient.

The availability of resource r can be estimated in two ways: with or without R. The resource state can be viewed as a ratio to the sum of values of r, or as ratios to each corresponding R.

The following template and its notation are specific to the Mathcad workspace.

The *cspline* evaluates the coefficients for the interpolating functions, the functions defined by *f* are the cubic spline functions of interpolation for species, and by *g* for the resources, with (gsas) or without (gsa) values for R.

z is the index of summation taking values from the minimum to the maximum value of the gradient x with a step selected by the user; here, we take a step (or lag) of 0.1.

Applying equation 7 to the interpolation functions described above leads to the value of HS when values of R are not available, or if they are, and considered in the research, the measure becomes HS_with_R, as given below (equations written as in Matchad template):

The Re( ) means that only the real part of the calculus is taken in cases when there is also an imaginary part that can be ignored by the researcher. Results are shown in Fig. 4.

**Fig. 4** Interpolation functions of resources’ use by two species (fsp1 and fsp2) and of the resource's availability with (gsas), and without (gsa), values of reference (R) in the environment, plotted against a continuous gradient (z)

These analyses repeated for *s* species will produce the niche similarity or overlap matrix that will be considered for further multivariate analyses as described in the article.

For cumulative functions (values for resources' use or availability), only areas delimited by a specific boundary will be considered. For instance, if relationships between resource use and availability are to be compared at different intervals of the gradient, the integrals from the formerly defined equations will contain boundaries (instead of min(x) and max(x)) defined by the research questions and demands.
